# Deep brain stimulation of the Cuneiform nucleus for levodopa-resistant freezing of gait in Parkinson’s disease: study protocol for a prospective, pilot trial

**DOI:** 10.1186/s40814-021-00855-7

**Published:** 2021-06-02

**Authors:** Stephano J. Chang, Iahn Cajigas, James D. Guest, Brian R. Noga, Eva Widerström-Noga, Ihtsham Haq, Letitia Fisher, Corneliu C. Luca, Jonathan R. Jagid

**Affiliations:** 1 The Miami Project to Cure Paralysis, Miami, FL USA; 2 Department of Neurosurgery, University of British Columbia, Vancouver, BC Canada; 3 Department of Neurological Surgery, University of Miami Miller School of Medicine, 1095 N.W. 14th Terrace, Miami, FL 33136 USA; 4 Department of Neurology, University of Miami Miller School of Medicine, Miami, FL USA

**Keywords:** Freezing of gait (FOG), Gait dysfunction, Parkinson’s disease, Mesencephalic locomotor region (MLR), Cuneiform nucleus (CnF), Pedunculopontine nucleus (PPN)

## Abstract

**Background:**

Freezing of gait (FOG) is a particularly debilitating motor deficit seen in a subset of Parkinson’s disease (PD) patients that is poorly responsive to standard levodopa therapy or deep brain stimulation (DBS) of established PD targets such as the subthalamic nucleus and the globus pallidus interna. The proposal of a DBS target in the midbrain, known as the pedunculopontine nucleus (PPN) to address FOG, was based on its observed pathology in PD and its hypothesized involvement in locomotor control as a part of the mesencephalic locomotor region, a functionally defined area of the midbrain that elicits locomotion in both intact animals and decerebrate animal preparations with electrical stimulation. Initial reports of PPN DBS were met with much enthusiasm; however, subsequent studies produced mixed results, and recent meta-analysis results have been far less convincing than initially expected. A closer review of the extensive mesencephalic locomotor region (MLR) preclinical literature, including recent optogenetics studies, strongly suggests that the closely related cuneiform nucleus (CnF), just dorsal to the PPN, may be a superior target to promote gait initiation.

**Methods:**

We will conduct a prospective, open-label, single-arm pilot study to assess safety and feasibility of CnF DBS in PD patients with levodopa-refractory FOG. Four patients will receive CnF DBS and have gait assessments with and without DBS during a 6-month follow-up.

**Discussion:**

This paper presents the study design and rationale for a pilot study investigating a novel DBS target for gait dysfunction, including targeting considerations. This pilot study is intended to support future larger scale clinical trials investigating this target.

**Trial registration:**

ClinicalTrials.gov identifier: NCT04218526 (registered January 6, 2020)

## Background and rationale

### Refractory freezing of gait in Parkinson’s disease

Gait dysfunction is a common and heterogeneous feature of Parkinson’s disease (PD), significantly impacting patients’ quality of life and increasing their risk for falls [1]. Common characteristics of parkinsonian gait include decreased stride length, reduced arm swing amplitude, and a flexed and stiffened trunk posture [2]. Some of these aspects of gait are improved with dopaminergic therapy or deep brain stimulation (DBS) of conventional targets such as the subthalamic nucleus (STN) or globus pallidus interna (GPi) [3]. Other deficits, such as postural instability and freezing of gait (FOG), may not respond or even worsen [4].

FOG is considered the most disabling of these gait deficits and is described as the transient and episodic failure to move forwards, despite intending to walk [5]. Patients describe feeling as if their feet are “glued” to the floor, and these episodes result in significantly decreased quality of life and contribute to falls and their related morbidities [6–8]. FOG is triggered in situations that highlight both cognitive and sensory influences on gait: during attempts to initiate stepping or turning, when navigating narrow corridors and obstacles, and when the patient is distracted or under duress [9, 10]. Conversely, these episodes frequently improve or resolve with the help of auditory or visual cues, such as a metronome set to a step cadence, or lines marked on the floor set to a desired stride length [11].

The pathophysiology of FOG is incompletely understood, but observations that it can be refractory to dopaminergic therapy suggest that it involves both dopaminergic and non-dopaminergic mechanisms [12]. FOG is associated with advanced PD [13], though it can present early [11]. While some controversy remains as to whether severe gait difficulties represent a distinct spectrum of the disease or merely a more advanced stage of the disease [14], many clinicians distinguish between tremor dominant (TD) and postural instability and gait difficulty dominant (PIGD) subtypes of PD, both in terms of their clinical prognoses as well as their management [15]. FOG is predominantly seen in the PIGD subtype and has an inconsistent response to levodopa or DBS. The development of an effective treatment for FOG remains a priority in the field.

### Rationale for DBS of the mesencephalic locomotor region

The mesencephalic locomotor region (MLR) is a physiologically defined area in the midbrain tegmentum, where electrical stimulation was found to initiate locomotion in cats [16, 17]. Since its discovery in 1966, the MLR has been identified in multiple species, as a phylogenetically preserved node in the supraspinal locomotor network [18–23], with studies suggesting therapeutic potential in animal models of gait disorders [24–26]. Electrophysiological and functional imaging evidence also supported its existence in humans [27–29], encouraging clinical interest in the region as a DBS target to promote gait. Specifically, this was thought to be a potential treatment for patients with the PIGD subtype of PD [15] and FOG that did not respond to levodopa therapy or conventional DBS of the STN or GPi [30–32].

Over the past 15 years, several centers have reported on DBS of the pedunculopontine nucleus (PPN), a putative anatomical component of the MLR, for postural instability and gait dysfunction in PD [33]. Despite initially promising case reports [34, 35], the efficacy of this therapy has since been called into dispute through the results of double-blinded studies and meta-analyses [36–39].

**Fig. 1:**
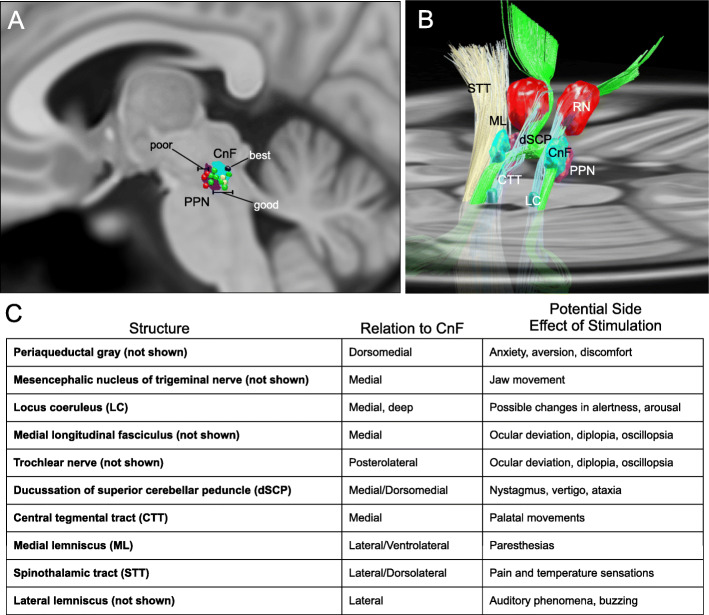
Three-dimensional anatomy of the human MLR and expected side effects of DBS. Reconstructions were made using Lead-DBS and available MNI-space subcortical atlases [44]. A separate CnF (cyan) NIfTI object was created in relation to the PPN (dark purple) based on Olszewski and Baxter’s Atlas [45]. **A** Sagittal projection (5 mm lateral to midline) of the CnF (cyan) and PPN (dark purple) with overlay of active contacts from PPN DBS patients with poor (red), good (green), best (blue), and unevaluated (yellow) gait outcomes from Goetz et al. [43]. **B** Diagonal 3D view with right ML and STT absent to visualize the MLR, projected on to a transverse slice of the brain at the level of the pons. **C** Chart lists nearby structures, their relation to the CnF, and expected side effects of stimulation. CnF Cuneiform nucleus, CTT central tegmental tract, dSCP decussation of superior cerebellar peduncle, LC locus coeruleus, ML medial lemniscus, PPN Pedunculopontine nucleus, RN red nucleus, STT spinothalamic tract. Adapted from Fig. 2 of [46] with permission

Several reasons have been posited to explain this lack of efficacy, including potential species differences in MLR function, and the degeneration of MLR neurons in PD [40–42]. A recent clinical study of DBS assessed FOG outcomes using a responder analysis. Of the reported subjects, there was a “good responder” cluster with near resolution of percent time spent in FOG with DBS on compared to off (34.1 ±14% vs. 2.7 ±2.6%) [43]. The best responders had active electrodes in the dorsal part of the MLR in the cuneiform nucleus (CnF) rather than the PPN (Fig. 1) [43]. This aligns with recent optogenetic studies in rodents, which showed that stimulation of glutamatergic neurons in the CnF effectively and specifically caused locomotor initiation [47, 48] (For review see [46]). It also agrees with preclinical electrophysiological studies in the cat, where stimulation of low threshold sites within the MLR primarily showed CnF neuron activation [49]. The importance of electrode targeting on efficacy in this region is reinforced by computer modeling studies, demonstrating that targeting errors of 1 mm can significantly decrease target activation selectivity [50].

Thus, this pilot study is designed to test the safety, feasibility, and preliminary efficacy of CnF DBS in alleviating FOG. Through this and future larger scale studies, we ultimately aim to explore the hypothesis that the CnF represents the neuroanatomic basis of the MLR and that suboptimal targeting may have played a role in the equivocal results of prior PPN DBS studies. We hope to confirm the findings in Goetz et al. [43], which suggest that minute changes in DBS target location in this area have significant impacts on clinical outcome and determine if CnF DBS may be a viable therapy for FOG and other gait dysfunctions.

## Methods/design

### Study design and overview

**Fig. 2:**
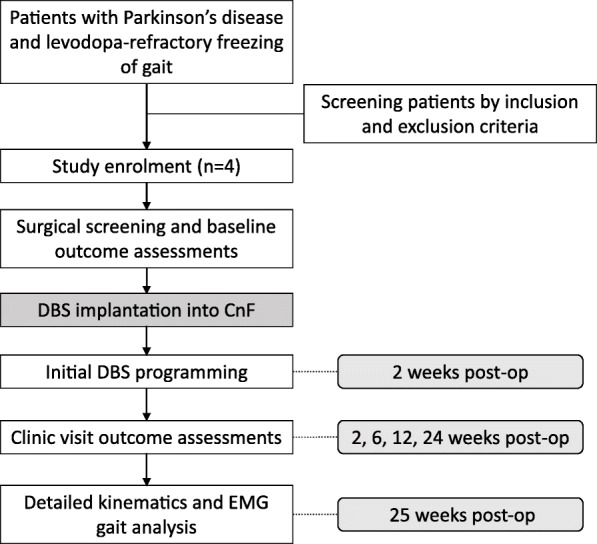
Flow chart of study outline

This study is a prospective, non-randomized, single-arm, open-label, pilot clinical trial designed to evaluate the safety and feasibility of delivering DBS to the CnF to alleviate freezing of gait in PD patients with severe, levodopa-resistant gait freezing. This study was approved by the University of Miami Human Subject Research Office (UM HSRO) and the US Food and Drug Administration with an Investigational Device Exemption as a phase I trial. The trial is registered in ClinicalTrials.gov: (NCT04218526). The study outline is shown in Fig. 2.

Suitable eligible patients will be screened for study participation and begin the consenting process, where the study objectives and the risks and benefits of participating are explained to them. Informed consent forms for the study will be filled out by subjects to confirm enrollment and they will undergo a baseline assessment, including a thorough neurological and neuropsychiatric examination, as well as standardized subjective and objective gait assessments. The Columbia Suicide Severity Rating Scale is included during screening given the known risk of suicidality with DBS for PD [51]. After enrolment and baseline evaluation, subjects will be consented for surgery prior to bilateral implantation of DBS electrodes in the CnF, with a post-operative CT scan to localize final electrode positions. For ethical reasons, all subjects will receive active stimulation beginning at 2 weeks post-implantation and will undergo repeated gait assessments in the clinic at 2-, 6-, 12-, and 24-weeks post implantation (Table 1). Gait assessment tests will be performed on medication, with DBS on and off, and assessors will be blinded to whether the DBS is on or off to minimize the risk of bias. A more detailed kinematics and EMG gait assessment, using the Nexus system (Vicon Motion Systems Ltd) will take place at study onset and conclusion. At study conclusion, subjects will decide whether they want to continue with stimulation or discontinue stimulation; in both cases, they will continue to receive their usual standard care and follow up with their neurologist.

**Table 1:** Detailed study schedule

Assessment	Visit 1	Visit 2	Visit 3	Visit 4	Visit 5	Visit 6	Visit 7	Visit 8	Visit 9
	Screen	Baseline assessment	Surgical screening	Surgery	2-week F/U	6-week F/U	12-week F/U	24-week F/U	25-week F/U
**Screening ICF**	X								
Pre-op work up^*^ **(CBC, CMP, β-hCG, INR/PTT/PT, U/A, EKG, CXR)**	X		X						
**Neuropsychiatric evaluation (including MDRS and BDI)**	X	X	X		X	X	X	X	
**Columbia Suicide Severity Rating Scale**	X	X	X		X	X	X	X	
**Gait assessment**	X	X			X	X	X	X	
**MDS-UPDRS**	X	X			X	X	X	X	
**FOG Questionnaire**		X			X	X	X	X	
**Timed Up and Go test**		X			X	X	X	X	
**Velocity test**		X			X	X	X	X	
**Variability test**		X			X	X	X	X	
**PDQ-39**		X	X		X	X	X	X	
**PDQL**		X	X		X	X	X	X	
**Brief pain history and NPSI**		X	X		X	X	X	X	
**Surgical ICF**			X						
**MRI**			X						
**Device implantation**				X					
**CT scan**				X					
**EMG analysis**		X		X					X
**Programming**					X	X	X	X	X
**Gait kinematics**		X							X

*BDI* Beck Depression Inventory, *β-hCG* Beta human chorionic gonadotropin, *CBC* complete blood count, *CMP* comprehensive metabolic panel, *CT* computed tomography, *CXR* chest x-ray, *EKG* electrocardiogram, *EMG* electromyogram, *FOG* freezing of gait, F/U follow-up, *INR* international normalized ratio, *NPSI* Neuropathic Pain Symptom Inventory, *MDRS* Mattis Dementia Rating Scale, *MDS-UPDRS* Movement Disorder Society Unified Parkinson’s Disease Rating Scale, *MRI* magnetic resonance imaging, *PDQ-39* Parkinson’s Disease Questionnaire-39, *PDQL* Parkinson’s Disease Quality of Life questionnaire, *PT* prothrombin time, *PTT* partial thromboplastin time, *U/A* urinalysis

^*^Pre-op labs are viable for 30 days before surgery. After 30 days, they must be repeated before surgical procedure

### Participants

We are actively screening and recruiting patients for this study from the Movement Disorders Clinic at the University of Miami Hospital. As this is a pilot study, we plan to enroll 4 patients with PD with levodopa-resistant FOG. A movement disorder neurologist will assess the eligibility of a patient based on the inclusion and exclusion criteria are detailed below.

### Inclusion criteria

- Age 40–75
- FOG is the primary complaint despite optimized dopaminergic therapy by a movement disorder neurologist
- Clinical observation of FOG in the ON/OFF states by a movement disorder neurologist
- PD stage 3 with good response to levodopa (defined as greater than 20% improvement in MDS-UPDRS score), except for severe gait disorder
- PD stage 3 with severe gait dysfunction and predominant axial symptoms defined as TD/PIGD ratio ≤ 0.90 (mean value of MDS-UPDRS items 2.10, 3.15a, 3.15b, 3.16a, 3.16b, 3.17a, 3.17b, 3.17c, 3.17d, 3.17e, and 3.18 divided by the mean value of MDS-UPDRS items 2.12, 2.13, 3.10, 3.11, and 3.12) [15] and FOGQ score> 12
- Minimal tremor, bradykinesia, and rigidity, or well controlled with levodopa.
- Agrees to full 6-month study participation

### Exclusion criteria

- Individuals with major executive dysfunction, dementia (Mattis Dementia Rating Scale-2 score ≤ 130), depression (Beck Depression Inventory II ≥ 25), or other neurocognitive impairments
- Presence of major medical co-morbidities and other surgical contra-indications
- Individuals requiring diathermy, transcranial magnetic stimulation, or electroconvulsive therapy
- Individuals with prior intracranial surgery
- Individuals with non-MRI compatible metallic implants in their head or active implantable devices anywhere in the body
- Individuals who are pregnant, breastfeeding, or the desire to become pregnant during the study
- Individuals on investigational drugs or any other intervention (not part of the guidelines for management of PD) known to have a potential impact on outcome

### Assessments

The following assessments will be completed to screen for study/surgical eligibility:

1. History and physical exam, including a neurological and clinical gait assessment
2. Blood pressure and postural drop
3. Pre-operative lab work, including 12-lead electrocardiogram and chest x-ray
4. Neuropsychiatric evaluation, including the Mattis Dementia Rating Scale-2, Beck Depression Inventory II, and the Columbia Suicide Severity Rating Scale

The following assessments will be completed prior to DBS implantation as well as at each clinic visit post-implantation:

1. MDS-UPDRS
2. FOG Questionnaire
3. Timed Up and Go (TUG) test
4. Instrumented gait assessment (Opal Inertial measurement unit, APDM Inc., Portland, OR) measuring:
  - Stride length (average over 2-min walk)
  - Velocity (average over 2-min walk)
  - Gait variability (measured over a 2-min walk)
5. 360° clockwise and counterclockwise turn on the spot (time and number of steps)
6. The Parkinson’s Disease Questionnaire (PDQ-39) and the Parkinson’s Disease Quality of Life Questionnaire (PDQL)
7. Columbia Suicide Severity Rating Scale
8. A brief pain history and Neuropathic Pain Symptom Inventory
9. Blood pressure and postural drop assessed in the off and on DBS stimulation state.

At study conclusion, patients will undergo a detailed kinematics gait assessment using the Nexus system (Vicon Motion Systems Ltd) and surface EMG recordings in the lower limbs.

### Device implantation

The Vercise™ (Boston Scientific Corporation) DBS System and Cartesia™ (Boston Scientific Corporation) directional leads will be used in this study. Implantation of electrodes and generator will be performed in one procedure, as previously described at our institution [52] but targeting the CnF. A pre-operative 3T MRI will be obtained prior to surgery. Subjects will be admitted on the day of surgery, and under intravenous sedation, a CRW frame will be placed and a CT obtained. The CT and MRI will be merged to obtain frame-based coordinates for the CnF. The default CnF coordinates will be calculated based on MRI brainstem landmarks to target brainstem normalized coordinates of (0.50, 0.25, 0) [43], and diffusion tractography will be used to ensure that our target is within the area demarcated by the medial lemniscus, superior cerebellar peduncle, and central tegmental tract. Trajectory planning will be performed to avoid vessels and ependymal and pial surfaces. Blood pressure and heart rate will be monitored by arterial line, and systolic blood pressure will be maintained between 90 and 120 mmHg during electrode insertions to reduce the risk of hemorrhage. The DBS lead placements will be preceded by microelectrode recordings and test stimulations to assess intraoperative physiology and rule out potential side-effects that may warrant repositioning of the lead (Fig. 1C). Intraoperative lower-extremity EMG will be used to assess for muscle activation to assist with targeting, as has been noted in animal studies targeting the CnF [49, 53]. If no such changes are noted, the lead positioning is determined only by target coordinates and avoiding side effects of stimulation. The use of an intraoperative O-arm (Medtronic) spin to provide real-time estimates of lead trajectory and location will be an option if there are any concerns about targeting or off-target stimulation effects. After implantation, patients will be fully anesthesized to undergo subcutaneous implantation of the generator in the chest to complete the procedure. Patients will be admitted to a post-surgical unit overnight for monitoring, including blood pressure and vital signs.

### DBS programming

Intraoperative stimulation parameters will be used to guide initial programming. Programming sessions will have heart rate, blood pressure, respiratory rate, and blood oxygenation saturation monitoring to mitigate the risk of adverse cardiorespiratory events related to stimulation. Additionally, low frequencies will initially be explored with patients until an adequate stimulation amplitude is found where gait initiation is under volitional control. Stimulation amplitude will then be increased until an adequate gait speed is achieved. If this is not immediately apparent, stimulation amplitude will be increased to just below the threshold for onset of side effects. Based on other studies, cyclic stimulation with continuous daily stimulation and night arrests will be the default protocol to avoid habituation and waning of effects [43, 54]. Current steering will be used to increase the therapeutic window and maximize chances of therapeutic benefit.

### Safety and feasibility

The analyses for safety will be descriptive, focusing on trends for within-subject differences and changes in FOG symptoms or off-target side-effects during the duration of the study. All adverse events (AEs) will be listed and their incidence compared to historical controls. The primary safety analysis will be conducted on all patient data when all 4 participants have completed the study.

### Endpoints of safety and feasibility

1. Death
2. Occurrence of pre-defined stoppage rules
3. Successful implantation and complete follow-up of 4 patients

Outcomes of neurological and functional status, gait, and quality-of-life are collected at several time points during the study (Table 1).

### Secondary outcomes

1. Percent change in FOG Questionnaire (FOGQ), Parkinson’s Disease Questionnaire (PDQ-39), and the Parkinson’s Disease Quality of Life Questionnaire (PDQL)
2. Percent change in Movement Disorders Society Unified Parkinson’s Disease Rating Scale (MDS-UPDRS) Part III across study visits relative to pre-operative baseline evaluation.
3. Percent change in number of falls (between baseline and final gait testing sessions, and on item 13 of UPDRS III)
4. Percent change in gait velocity with and without CnF DBS
5. Percent change in muscle electromyogram (EMG) amplitude during gait testing at study conclusion between CnF DBS on and off conditions

### Kinematic and EMG evaluation of gait

Lower-limb gait kinematics and EMG will be evaluated with and without DBS in study subjects (*n* = 4). Subjects will be fitted with fifteen reflective markers that will be tracked at 100 Hz with a 10-camera Nexus system (Vicon Motion Systems Ltd) attached bilaterally to the first and fifth distal phalange of the foot, lateral malleolus, calcaneus, lateral femoral epicondyle, anterior superior iliac spine, and the mid-shank and mid-thigh. A reflective marker will also be placed on the lower back region overlying the sacrum. EMG surface electrodes will be placed over the tibialis anterior (TA) as well as the lateral gastrocnemius (LG). Subjects will be asked to walk along a 25-foot path at a constant, regular walking pace both without and then with optimal DBS configuration settings (CnF L on/CnF R on), DBS OFF (CnF L off/CnF R off), DBS RIGHT Only (CnF L off/CnF R on), and DBS LEFT only (CnF L on/CnF R off) repeating each condition twice. Kinematic and EMG signals will be recorded during each trial while the subjects walk for 25 ft. and averaged for each test condition.

### Handling of missing data

Because of the 25-week follow-up, efforts will be made to minimize the number of participants lost to follow-up by developing good rapport, making the participant feel comfortable with the research staff and having regular correspondence between assessments. To maintain contact and continued willingness for study participation at each visit participants will provide their current address and phone number and e-mail address as well as contact information for at least two individuals who live outside of their household yet are likely to know their whereabouts. Contact information and contact history will be entered into an electronic database allowing regular review and update.

Although every attempt will be made to avoid missing outcome data, missing data is anticipated with any longitudinal study. The missing data can be defined as intermittent or dropout. The reason for missing data will also be recorded and fully examined to confirm this assumption and to minimize this occurrence in any future confirmatory trials. Where appropriate, non-negligible missing data will be imputed using regression methods.

### Data analysis

The primary goal of this pilot study is to assess the safety and feasibility of delivering DBS to the CnF in PD patients with refractory FOG and thus will only involve 4 subjects. We believe this number of subjects will provide adequate data on safety and feasibility for a larger study. However, assessment of trend toward efficacy will be performed for the secondary outcomes. One-way ANOVA will be used to compare percent improvement in gait velocity for the four different DBS configurations (L CnF off/R CnF off, L CnF off/R CnF on, L CnF on/R CnF off, L CnF on/R CnF on) at each clinic visit, with the Tukey Honest Significant Difference post hoc test to compare configurations. The same test will be used to compare changes in EMG amplitude between the four DBS configurations at the final gait assessment at the study’s conclusion. One-way ANOVA with repeated measures will be used to compare changes in the UPDRS III, FOG Questionnaire, PDQ-39, and PDQL over the multiple time points of the study.

### Data management

All study documents and files will use an anonymous study identification number to identify subjects and only an appointed Study Coordinator will maintain linkages, to maintain subject confidentiality. Additionally, locally stored computer data will be password protected and located within our institution-based firewall. Physical study data files including signed consent forms will be stored securely in the principal investigator’s (JJ) office within a locked filing cabinet. Subject data will be documented in source documents initially and then recorded on electronic case report forms (CRFs). The Study Coordinator will ensure that data is entered into CRFs within 5 days of data collection and will review the accuracy of entered data by comparison with subjects’ medical records within 30 days. Potential discrepancies will be flagged and checked and corrected as necessary by study investigators.

Only personnel listed on study protocols will have access to study data, though study data that could medically benefit subjects will be shared with them. There will be periodic audits (at least two during the study) of the data by university’s Office of Research Compliance and Quality Assurance (RCQA) auditors to ensure compliance with the FDA’s Good Clinical Practices and ICH-E6. The Principal Investigator will submit all de-identified CRFs to the Study Sponsors and the FDA throughout and at the completion of the study, including any incomplete CRFs and CRFs of those who withdraw before study completion.

A Data Safety and Monitoring Board (DSMB) has been established as an independent expert advisory group to assess adverse events (AEs) and evaluate the general integrity and conduct of the study. The DSMB chair will be provided a monthly update on the status of all study subjects. Furthermore, all AEs related to the study will be reported to the DSMB, which will make recommendations as to whether the study should continue without change, be modified, or be terminated early. Pre-determined stoppage rules have also been established to aid with early termination decision making (Table 2). No formal interim analysis of efficacy is planned given the small number of participants.

**Table 2:** Pre-determined study stoppage rules

1	Occurrence of one or more SAEs with unexplained etiology and unsatisfactory resolution
2	Occurrence of one or more persistent and debilitating stimulation-related AEs in the CnF
3	Occurrence of hemorrhage, stroke, or paralysis related to device
4	Occurrence of changes in blood pressure, heart rate, and/or respiratory rate related to device function that occur outside of the clinic and require medical intervention
5	Significant worsening of any symptom of PD, other than transiently, including tremor, bradykinesia, rigidity, or gait that would not otherwise have been expected as part of the natural course of the disease

*AE* adverse event, *CnF* cuneiform nucleus, PD Parkinson’s disease, SAEs serious adverse event

## Discussion

PD patients with FOG that is refractory to dopaminergic therapy have limited therapeutic options. Our study protocol aims to test the safety, feasibility, and preliminary efficacy of a novel brainstem target to alleviate FOG. Our strategy is based on a careful revisiting of classical animal studies [16], newly emerging optogenetics data [47], and a recent clinical study [43], all of which suggest that optimizing the location of DBS within the MLR could significantly improve the effect of DBS on this gait network. While some have pointed to the inconsistent outcomes of previous PPN DBS studies as a reason to abandon this approach altogether [41], that subsets of patients with FOG show significant improvement with DBS in this region suggests that refinement of methodology may be important. Through this and future larger scale studies, we hope to determine if CnF DBS could be an effective therapy for FOG and gait dysfunction in other neurodegenerative disorders.

A strength of this study is the novel use of directional electrodes to deliver DBS to this midbrain region. As a complex area comprised of numerous interleaving fiber tracts and structures, the ability to steer current would be important in optimizing therapeutic effects while mitigating stimulation-induced side effects. Limitations of this study include the small sample size, given the preliminary nature of this study, and the lack of a control cohort. Additionally, as some patients with DBS in this region feel mild paresthesias with stimulation, complete double blinding of DBS status during gait assessments may be impossible. Finally, there are limitations related to the studying of FOG itself, including potential heterogeneity of pathobiology, and difficulty in eliciting FOG in the clinical setting. We hope to mitigate these difficulties through our rigorous inclusion/exclusion criteria, and by using known triggers for FOG during gait assessments, if necessary.

### Trial status

Study protocol version 2 was approved March 2, 2020, by the FDA. Recruitment started April 27, 2020, but was delayed due to the COVID-19 pandemic and is expected to be completed by December 31, 2022.

## Data Availability

All data in the study protocol are included in this article and supplementary files. Data from the study are unavailable at the time of publication. Once completed, dissemination of non-identifiable study results will occur through publication in a peer-reviewed journal, presentations, and seminars, irrespective of outcomes. Participants will be able to request a copy of study results from the Principal Investigator.

## Abbreviations

AE: Adverse event
CT: Computed tomography
CRF: Case report form
CnF: Cuneiform nucleus
DBS: Deep brain stimulation
DSMB: Data Safety Monitoring Board
EMG: Electromyogram
FOG: Freezing of gait
FTD: Frontotemporal dementia
GPi: Globus pallidus interna
LFP: Local field potential
MDS-UPDRS: Movement Disorder Society Unified Parkinson’s Disease Rating Scale
MER: Microelectrode recordings
MLR: Mesencephalic locomotor region
MRI: Magnetic resonance imaging
PAG: Periaqueductal gray
PD: Parkinson’s disease
PIGD: Postural instability and gait difficulty
PPN: Pedunculopontine nucleus
STN: Subthalamic nucleus
